# Supplementary material for: MethylBERT enables read-level DNA methylation pattern identification and tumour deconvolution using a Transformer-based model
Source: Nat Commun. 2025 Jan 17;16:788. doi: 10.1038/s41467-025-55920-z (PMC11742067; doi:10.1038/s41467-025-55920-z)
Supplement: Supplementary file 1 — Supplementary Information [file 41467_2025_55920_MOESM1_ESM.pdf]

Supplementary tables and figures

	HMM/Bayesian/Random Forest-based model	CNN/RNN-based model	Transformer-based model
**Methylation site prediction**	iDNA-MS<br>SOMM4mC<br>MM-6mAPred	DeepTorrent<br>iIM-CNN	iDNA-ABT<br>MuLan-Methyl
**Nanopore methylation calling**	Nanopolish<br>SignalAlign	DeepMod<br>DeepSignal	Rockfish<br>methBERT
**Single-cell methylation imputation**	Melissa<br>CaMelia	DeepCpG	CpG Transformer
**Tumour purity estimation**	CancerDetector<br>BED<br>DXM<br>CAMDAC	DISMIR	**<u>MethylBERT</u>**

Supplementary Table 1. Overview of methods for sequencing-based DNA methylome analysis

	CancerDetector	DISMIR	DISMIR_dmr	Houseman
**MethylBERT**	1.089e-09	2.594e-19	1.856e-03	4.013e-26
**MethylBERT +adjustment**	1.433e-09	1.102e-18	7.695e-04	2.392e-18

Supplementary Table 2. Two-sided paired t-test p-values with Bonferroni correction for Figure 4A. The p-value for MethylBERT vs MethylBERT+adjustment is 1.737e-04.

	CancerDetector	DISMIR	DISMIR_dmr	Houseman
**MethylBERT**	1.000e+00	8.828e-04	4.376e-09	1.370e-06
**MethylBERT +adjustment**	1.000e+00	1.228e-03	1.325e-08	1.451e-06

Supplementary Table 3. Two-sided paired t-test p-values with Bonferroni correction for Figure 5A. The p-value for MethylBERT vs MethylBERT+adjustment is 5.218e-01.

Cell type	Samples
Prostate epithelial	Prostate-Epithelial-Z000000RV<br>Prostate-Epithelial-Z000000S3<br>Prostate-Epithelial-Z0000045F<br>Prostate-Epithelial-Z0000045G
Non-prostate epithelial	Blood-T-EffMem-CD4-Z00000416<br>Colon-Fibroblasts-Z0000042C<br>Blood-B-Z000000UB<br>Blood-T-CD8-Z000000U5<br>Blood-B-Mem-Z0000041J

Supplementary Table 4. Normal cell-type methylation atlas samples used for training MethylBERT to estimate prostate epithelial cell-type proportions in Figures 4F and G.

Cell type	Samples
Blood-B	Blood-B-Z000000UB<br>Blood-B-Z000000UR
Blood-NK	Blood-NK-Z000000UF<br>Blood-NK-Z000000U1
Blood-Granul	Blood-Granulocytes-Z000000TZ<br>Blood-Granulocytes-Z000000UT
Blood-T	Blood-T-Eff-CD8-Z0000041Q<br>Blood-T-CenMem-CD4-Z0000041D
Blood-Mono+Macro	Blood-Monocytes-Z000000TP<br>Lung-Alveolar-Macrophages-Z00000448

Supplementary Table 5. Normal cell-type methylation atlas samples (Loyfer et al. [24]) used for training MethylBERT to deconvolute leukocyte samples in Figure 4H.

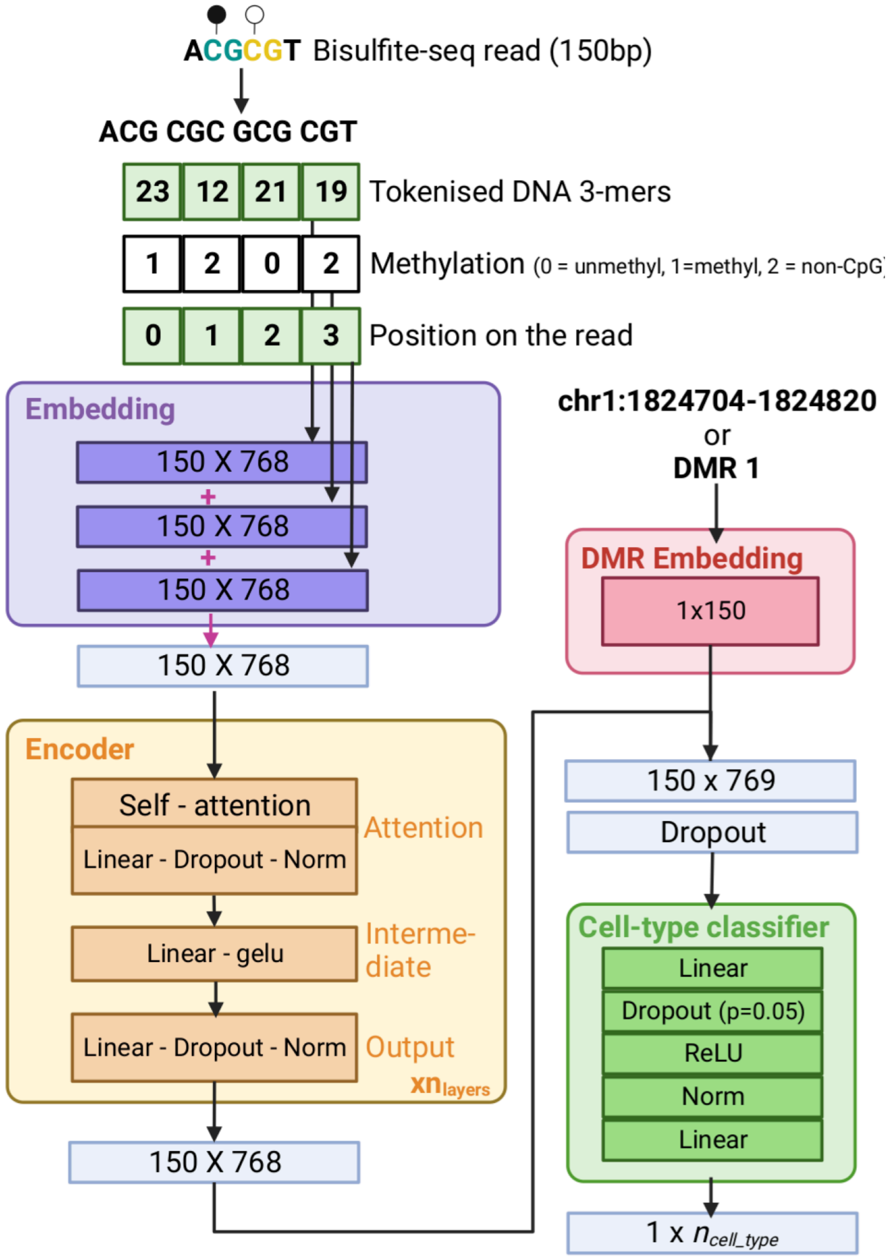

Supplementary Figure 1. MethylBERT network architecture

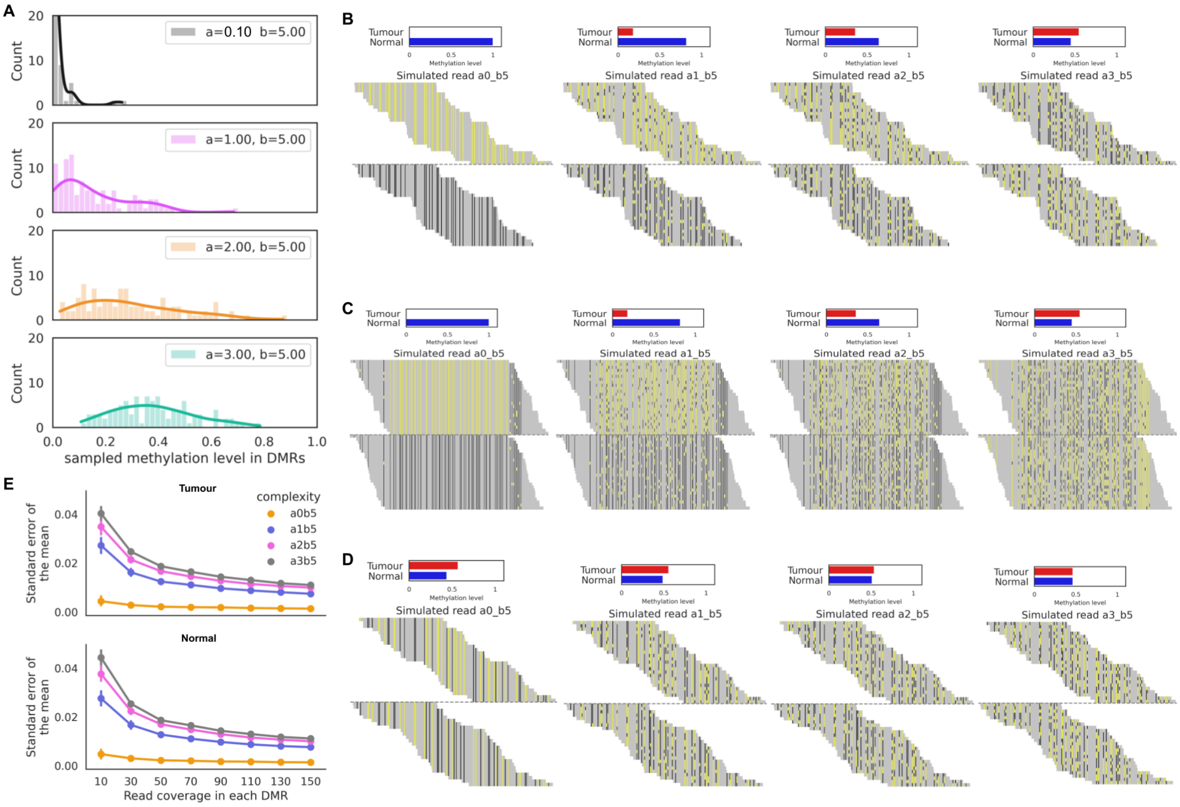

Supplementary Figure 2. Simulated read-level methylomes **A.** Distribution of tumour methylation level in DMRs (n=100) sampled from a beta distribution with different α values. **B-D.** Examples of simulated methylation patterns in four different complexities. The read length is set up as 150bps (B) and 500 bps (C), respectively. CpG-specific methylation patterns are given instead of region-specific methylation levels (D). The grey dotted line separates tumour methylation patterns (above the line) and normal methylation patterns (below the line). The histogram at the top of the methylation patterns indicates tumour and normal methylation levels in the region. **E.** Mean-variance of methylation patterns in DMRs for different coverage of simulated data. The error bars indicate a 95% confidence interval.

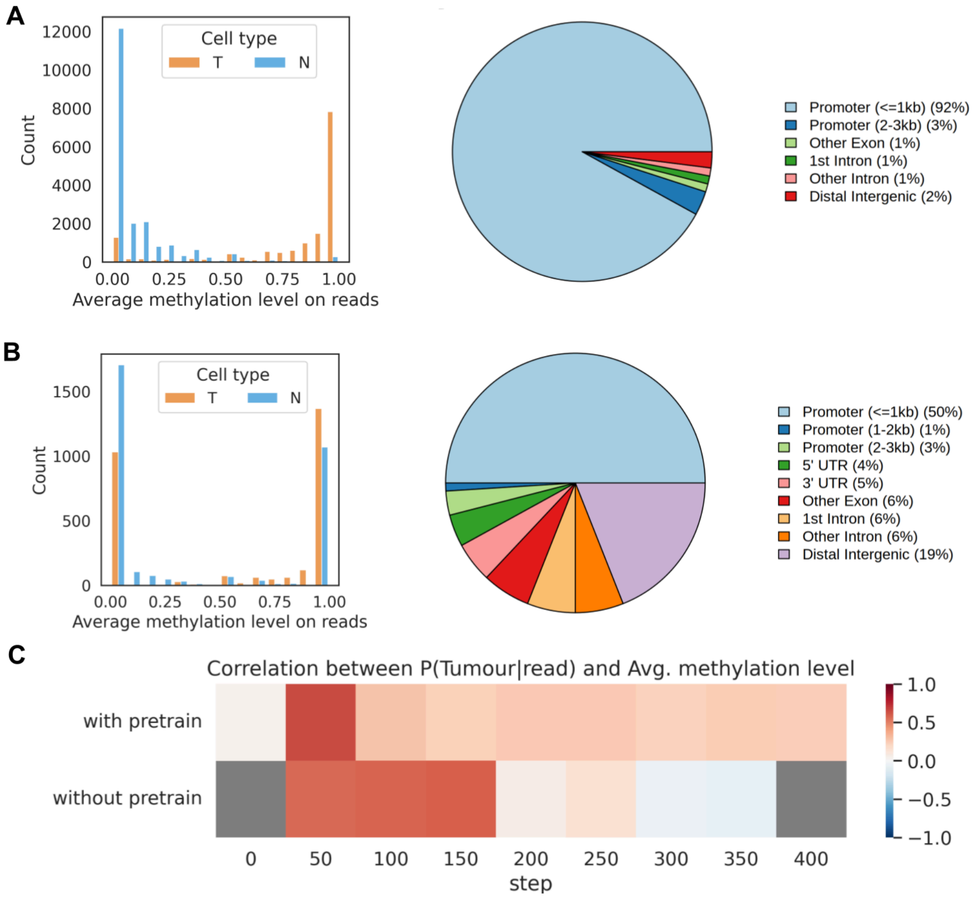

Supplementary Figure 3. Additional pre-training result analyses. **A.** Distribution of read-wise methylation level in DMRs selected according to areaStat score (left, n=34,881) and genomic annotation of DMRs (right, n=100). **B.** Distribution of read-wise methylation level in DMRs where hypomethylated and hypermethylated patterns are balanced (left, n=6,041) and genomic annotation of DMRs (right, n=100). **C.** Correlation between P(cell type=Tumour|read) and average methylation level in each DMR set before and after pre-training. Grey colour means an insignificant correlation value (p-value> 0.05).

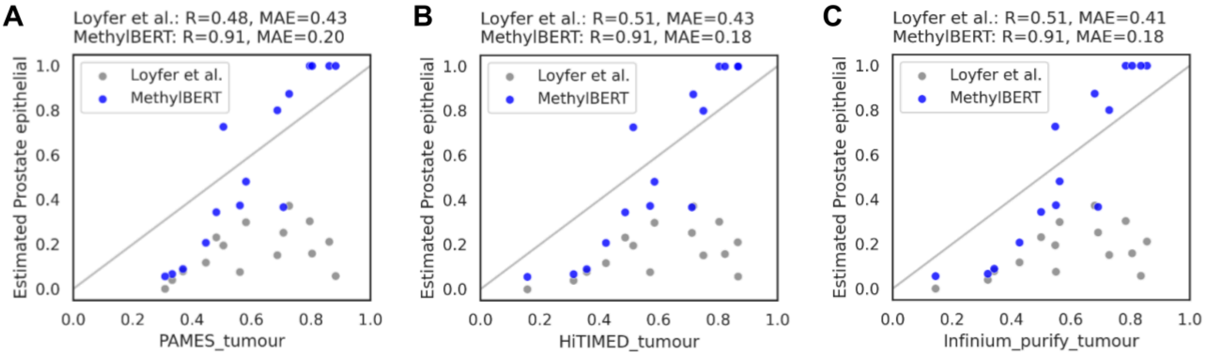

Supplementary Figure 4. Correlation between the estimated prostate epithelium proportion (without tumour reference data, by MethylBERT and Loyfer et al.'s method, respectively) and the estimated tumour purity using tumour reference data by PAMES (A), HiTIMED (B), and InfiniumPurify (C) for the lymph node samples (n=15) acquired from hormone-sensitive metastatic prostate cancer patients. Different methods are distinguished by different colours.

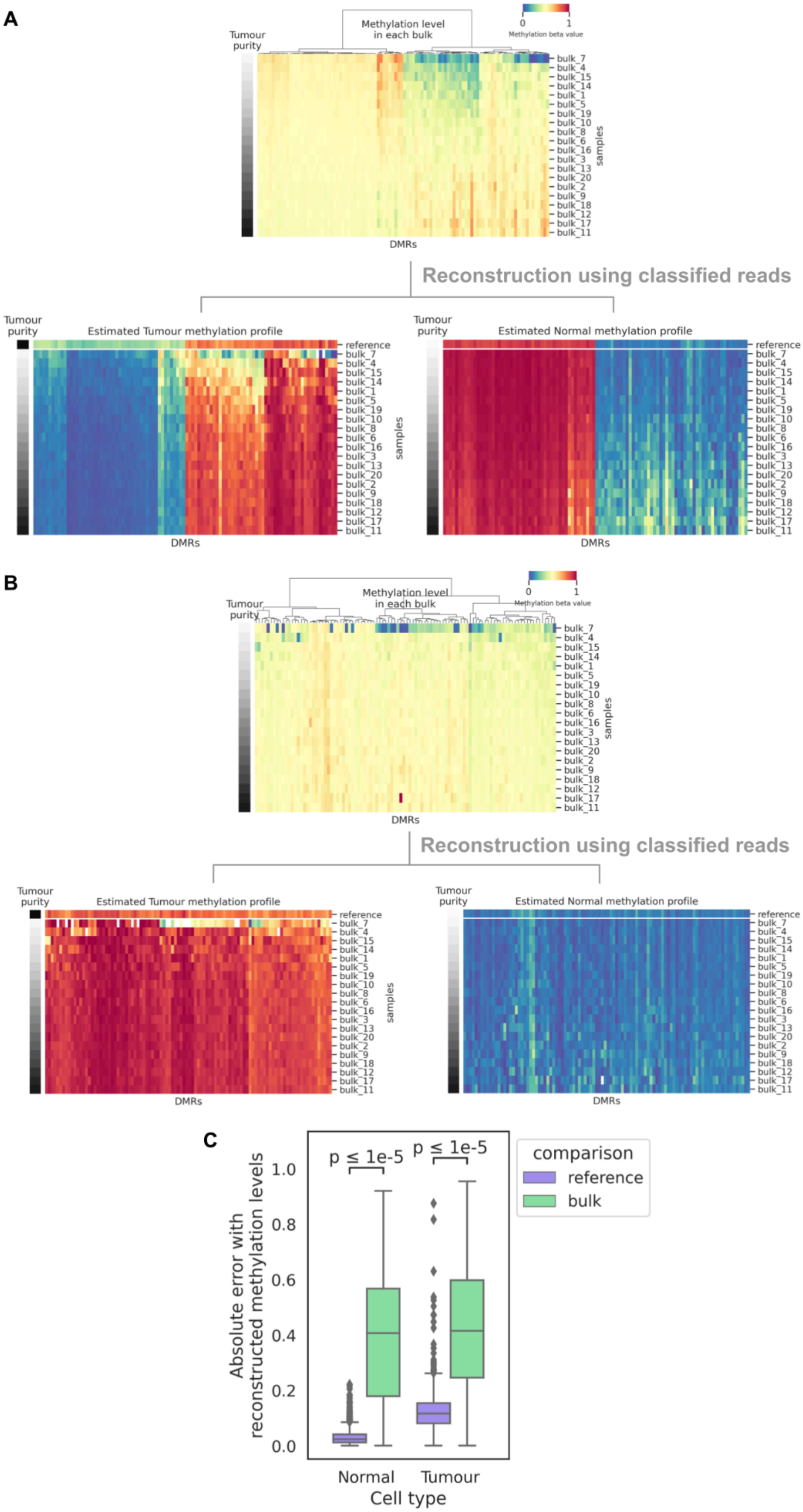

A
Methylation level
in each bulk
0
1
Methylation beta value
Tumour
purity
bulk_7
bulk_4
bulk_15
bulk_14
bulk_1
bulk_5
bulk_19
bulk_10
bulk_8
bulk_6
bulk_16
bulk_3
bulk_13
bulk_20
bulk_2
bulk_9
bulk_18
bulk_12
bulk_17
bulk_11
samples
DMRs
Reconstruction using classified reads
Tumour
purity
Estimated Tumour methylation profile
reference
samples
DMRs
Tumour
purity
Estimated Normal methylation profile
reference
samples
DMRs
B
Methylation level
in each bulk
0
1
Methylation beta value
Tumour
purity
samples
DMRs
Reconstruction using classified reads
Tumour
purity
Estimated Tumour methylation profile
reference
samples
DMRs
Tumour
purity
Estimated Normal methylation profile
reference
samples
DMRs
C
p ≤ 1e-5
p ≤ 1e-5
comparison
reference
bulk
Absolute error with
reconstructed methylation levels
1.0
0.8
0.6
0.4
0.2
0.0
Normal
Tumour
Cell type

Supplementary Figure 5. Reconstructed DMR methylation levels in tumour and normal cell type using the MethylBERT read classification results. **A-B.** Reconstructed cell type-specific methylation patterns from all pseudo-bulks compared to reference cell type-specific methylation profiles in DMRs, when DMRs are evenly divided between tumour-hypermethylated and -hypomethylated regions (A) and are selected based on areaStat values (B). The first row of two heatmaps at the bottom presents the reference methylation profile of the tumour and normal cell types. Bulks are ordered by the tumour purity and the original methylation beta-values of the bulks are shown in the top heatmap. **C.** Absolute error between reconstructed and reference methylation levels (purple), and between reconstructed and bulk methylation levels (green) for 100 regions selected based on areaStat (total 20 bulks). Statistics were calculated using paired t-test with Bonferroni correction.

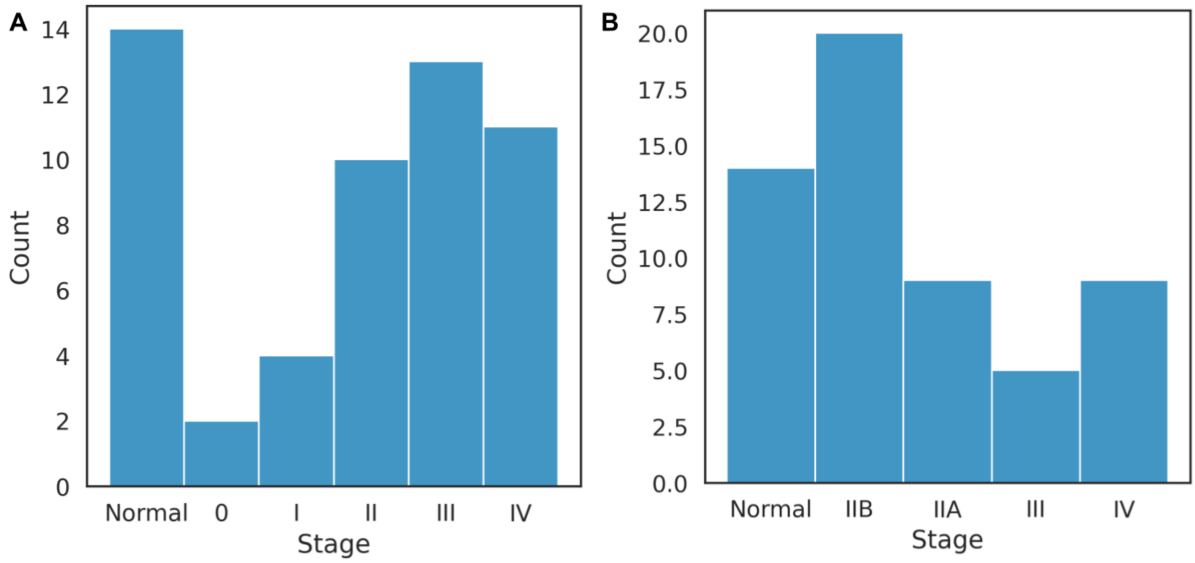

Supplementary Figure 6. Age histogram of cancer patients by stages in ctDNA samples from CRC patients (A) and from PDAC patients (B).

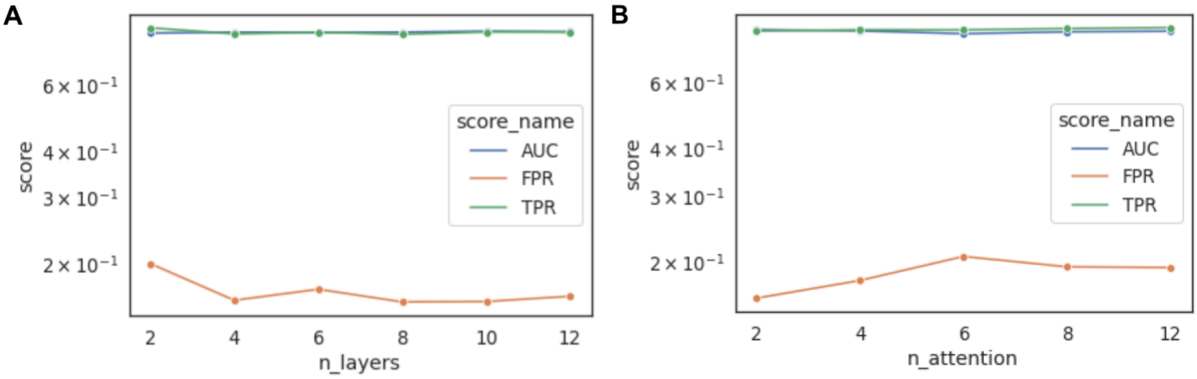

Supplementary Figure 7. Comparison of read-level methylome classification performance across different numbers of encoder layers (A) and different numbers of attention heads (B).

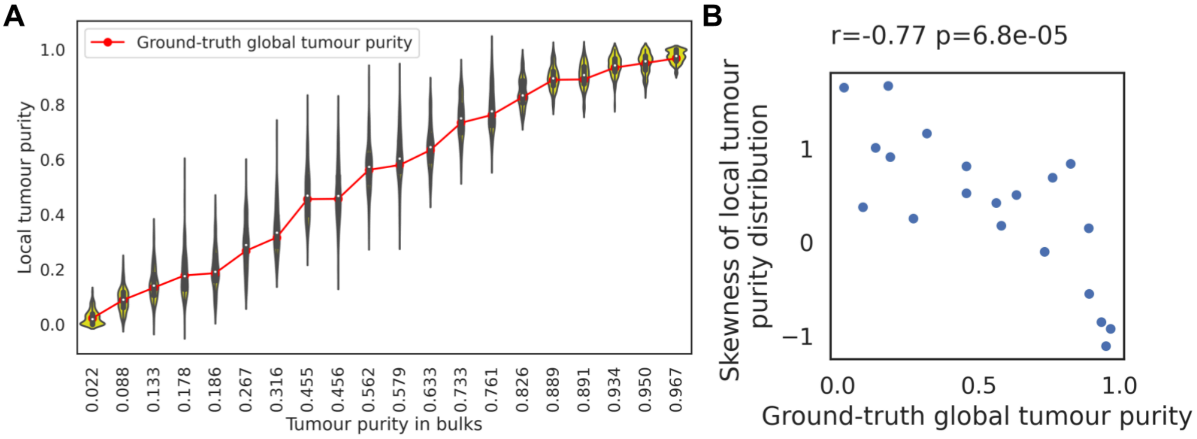

Supplementary Figure 8. Region-wise tumour purity in DLBCL pseudo-bulk samples. **A.** Distribution of region-wise tumour purity (local tumour purity) is presented in the violin plot. The ground-truth tumour purity of bulks is shown in the red line. The samples are ordered by the ground-truth value. The inner boxplots represent the median, and the first and third quartiles, whereas the whiskers show the rest of the distribution. **B.** Correlation between the skewness of the local tumour purity values and the ground-truth tumour purity. Two-sided p-value represents the probability that the absolute correlation coefficient of a random sample from an uncorrelated population ≥ the absolute value of a given correlation coefficient, as implemented in [23].

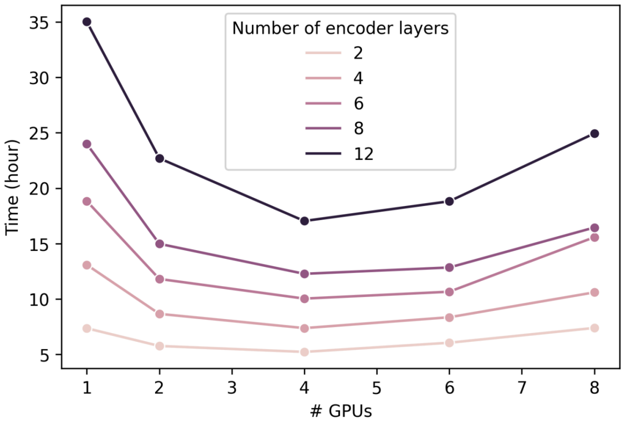

Supplementary Figure 9. Running time of MethylBERT by the number of GPUs and the number of encoder layers. Each run was done over 1000 steps.

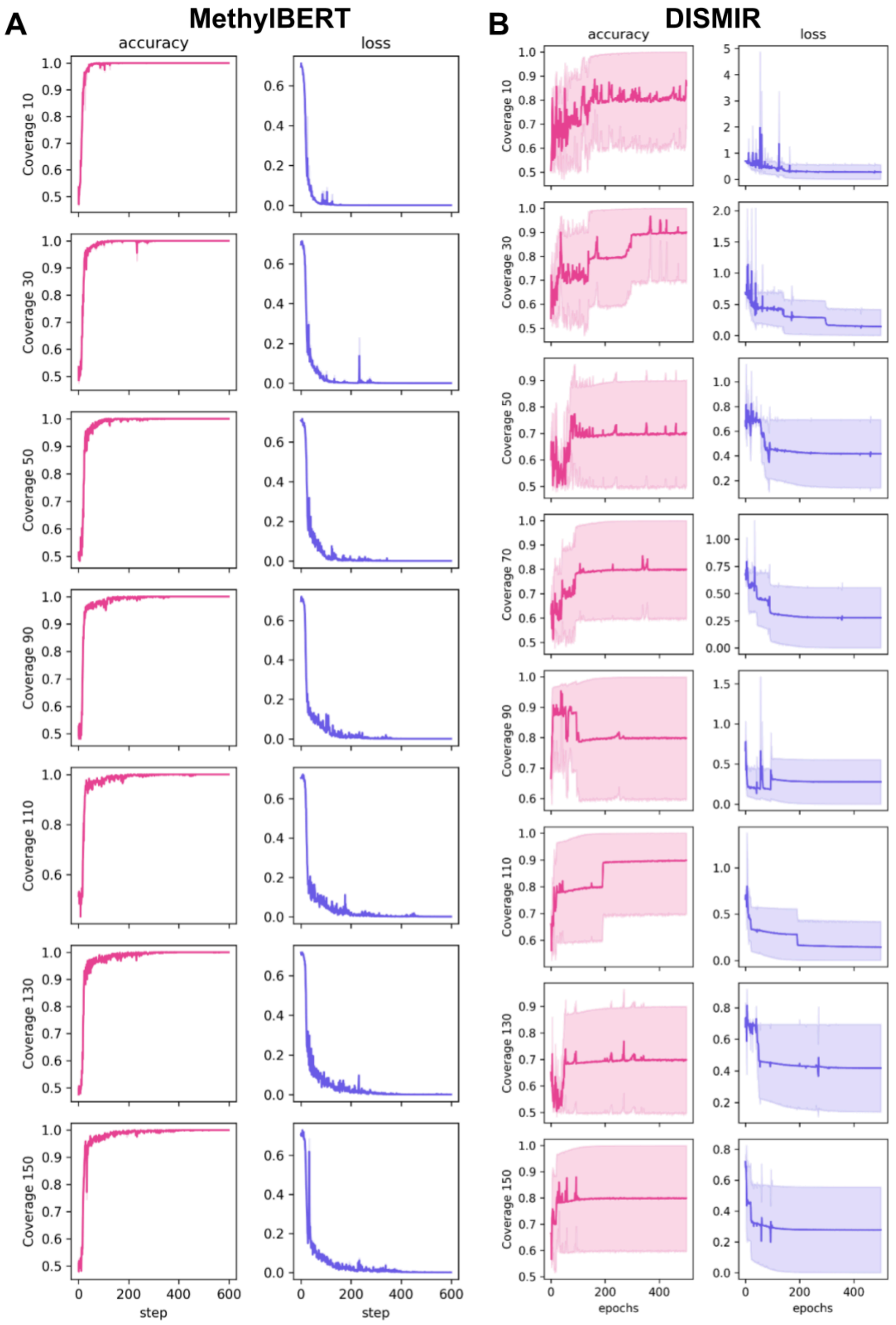

Supplementary Figure 10. Read classification accuracy and loss values for different read coverage (in the training set) yielded by MethylBERT (A) and DISMIR (B). We performed five times of the same training for both methods and the line plots show the 95% confidence interval and the mean over the five training runs.

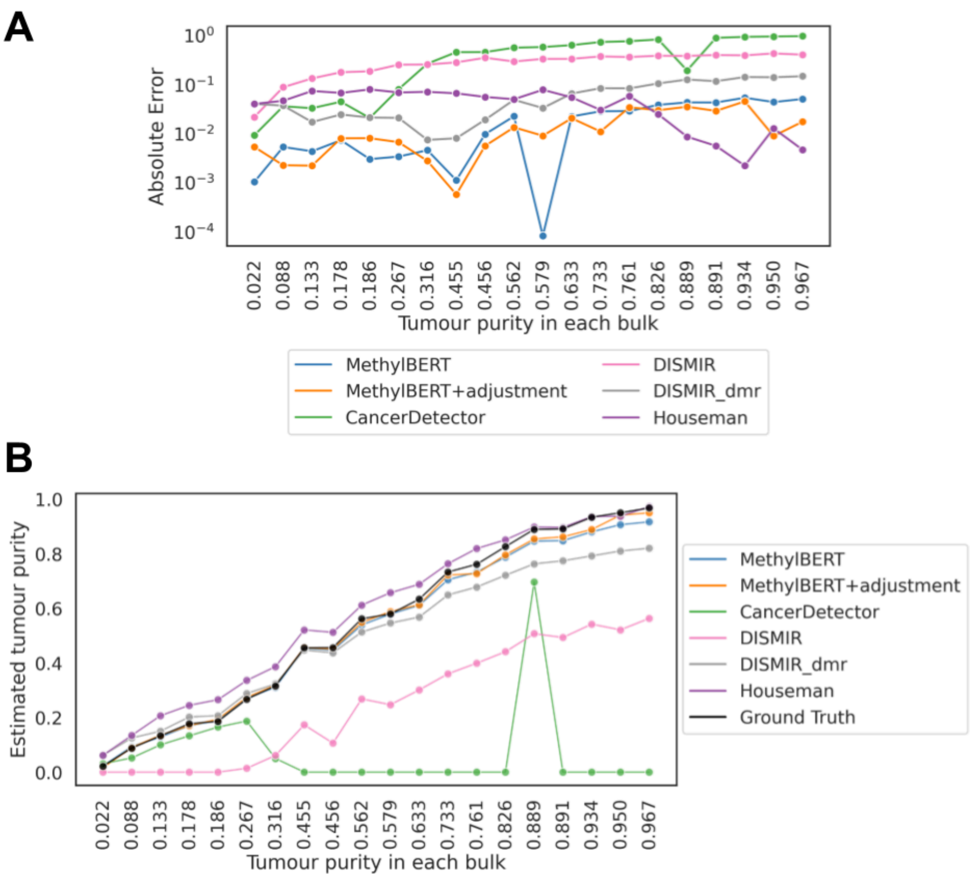

Supplementary Figure 11. Tumour purity estimation results for DLBCL pseudo-bulk samples. (A) Absolute error between the ground-truth and estimated tumour purities for each bulk. (B) Estimated tumour purities by individual methods and the ground-truth values for each bulk. Bulks are sorted according to the tumour purity on the x-axis in both plots.

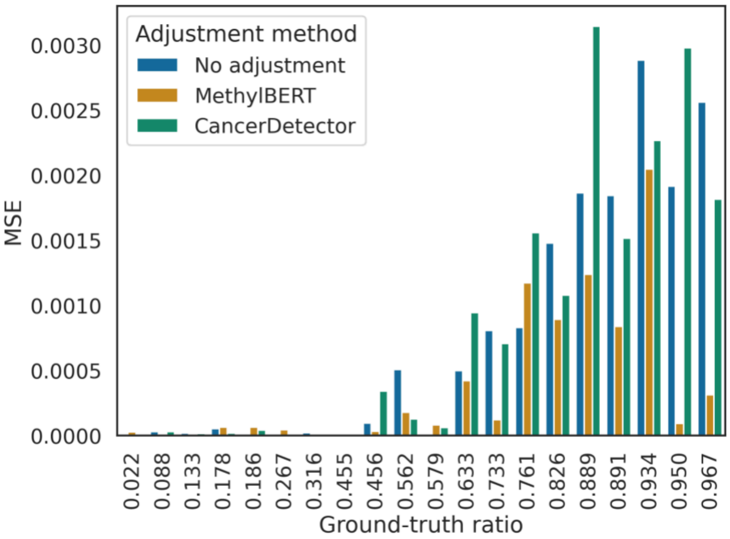

Supplementary Figure 12. Performance comparison of different estimation adjustment methods. The histogram shows the mean squared error between the ground-truth and the estimated tumour purity for pseudo-bulks sorted by the ground truth.

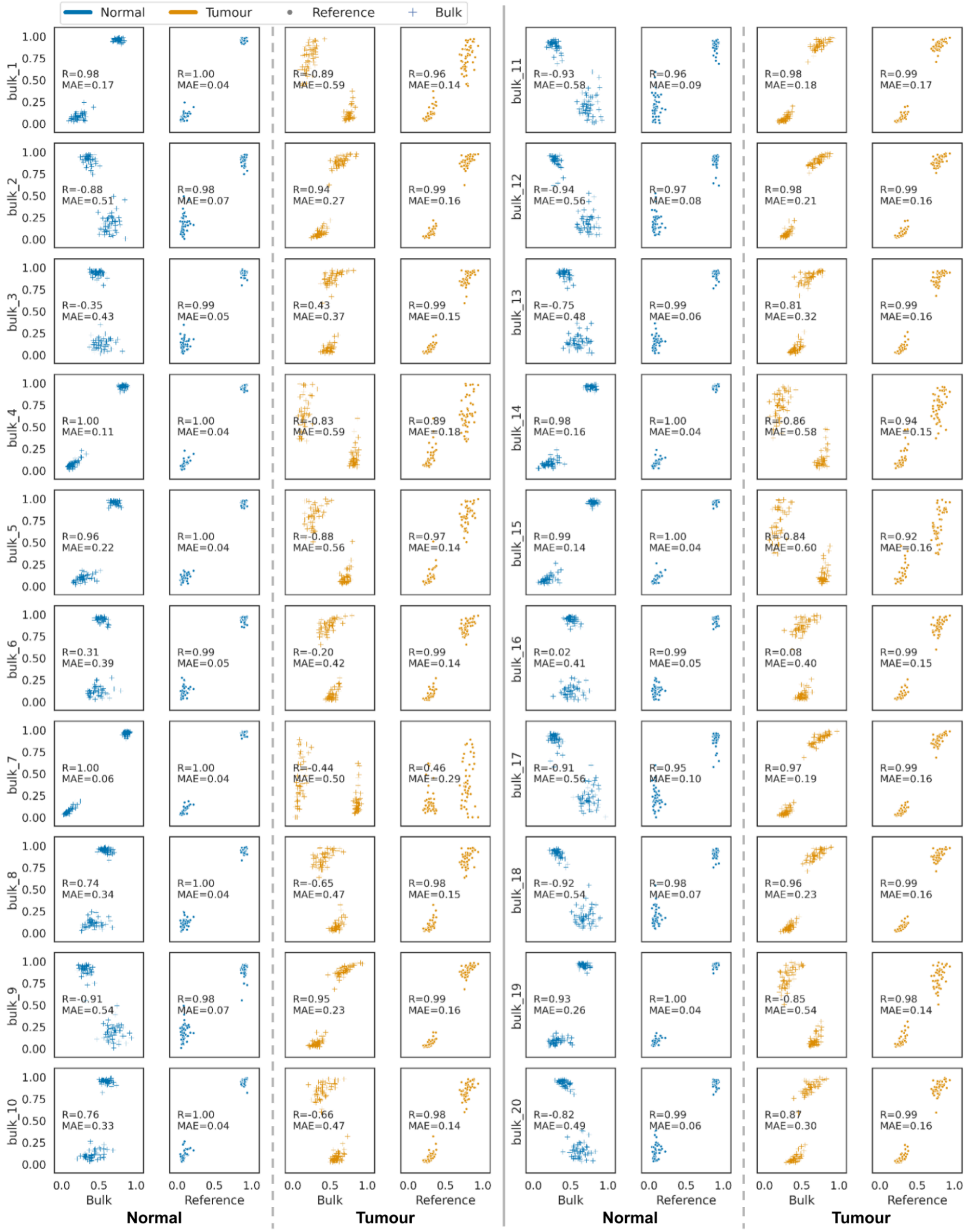

Supplementary Figure 13. Reconstructed cell type-specific methylation levels in DMRs (y-axis) compared to the bulk methylation profile (+ marker, x-axis) and the reference cell-type methylation profile (● marker, x-axis). Half tumour-hypermethylated and half tumour-hypomethylated regions were selected. Blue and orange colour indicates the normal and tumour cell types, respectively. In each analysis, mean absolute error (MAE) and Pearson correlation (R) were calculated.

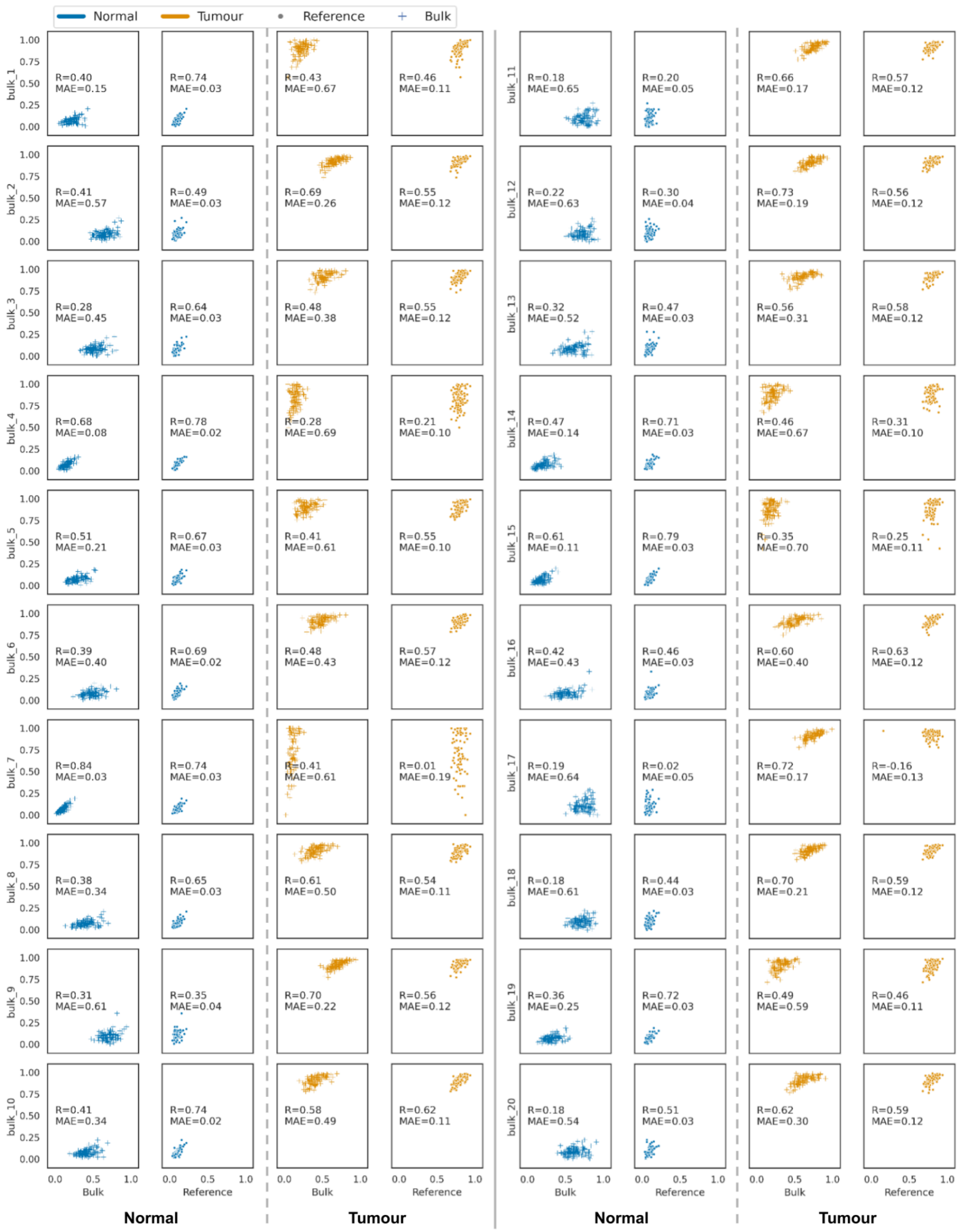

Supplementary Figure 14. Reconstructed cell type-specific methylation levels in DMRs (y-axis) compared to the bulk methylation profile (+ marker, x-axis) and the reference cell-type methylation profile (● marker, x-axis). 100 regions with the highest areaStat score were selected. Blue and orange colour indicates the normal and tumour cell types, respectively. In each analysis, mean absolute error (MAE) and Pearson correlation (R) were calculated.
