# Supplementary material for: MethylBERT enables read-level DNA methylation pattern identification and tumour deconvolution using a Transformer-based model
Source: Nat Commun. 2025 Jan 17;16:788. doi: 10.1038/s41467-025-55920-z (PMC11742067; doi:10.1038/s41467-025-55920-z)
Supplement: Supplementary file 3 — Reporting Summary [file 41467_2025_55920_MOESM3_ESM.pdf]

nature portfolio

Corresponding author(s): Pavlo Lutsik, Yunhee Jeong

Last updated by author(s): Nov 18, 2024

Reporting Summary

Nature Portfolio wishes to improve the reproducibility of the work that we publish. This form provides structure for consistency and transparency in reporting. For further information on Nature Portfolio policies, see our Editorial Policies and the Editorial Policy Checklist.

Statistics

For all statistical analyses, confirm that the following items are present in the figure legend, table legend, main text, or Methods section.

n/a	Confirmed	
☐	☒	The exact sample size (*n*) for each experimental group/condition, given as a discrete number and unit of measurement
☐	☒	A statement on whether measurements were taken from distinct samples or whether the same sample was measured repeatedly
☐	☒	The statistical test(s) used AND whether they are one- or two-sided<br>*Only common tests should be described solely by name; describe more complex techniques in the Methods section.*
☐	☒	A description of all covariates tested
☐	☒	A description of any assumptions or corrections, such as tests of normality and adjustment for multiple comparisons
☐	☒	A full description of the statistical parameters including central tendency (e.g. means) or other basic estimates (e.g. regression coefficient) AND variation (e.g. standard deviation) or associated estimates of uncertainty (e.g. confidence intervals)
☐	☒	For null hypothesis testing, the test statistic (e.g. *F*, *t*, *r*) with confidence intervals, effect sizes, degrees of freedom and *P* value noted<br>*Give P values as exact values whenever suitable.*
☐	☒	For Bayesian analysis, information on the choice of priors and Markov chain Monte Carlo settings
☒	☐	For hierarchical and complex designs, identification of the appropriate level for tests and full reporting of outcomes
☐	☒	Estimates of effect sizes (e.g. Cohen's *d*, Pearson's *r*), indicating how they were calculated

Our web collection on statistics for biologists contains articles on many of the points above.

Software and code

Policy information about availability of computer code

Data collection	Most of the data in the manuscript are publicly available and was downloaded from repositories in processed form. Wherever necessary raw sequencing data was preprocessed using the following tools:<br>Cutadapt v2.6<br>TrimGalore v0.6.6<br>Bismark v0.22.3<br>Mark Duplicates v1.141<br>samtools v1.9
Data analysis	This manuscript presents computational method and package MethylBERT that was used for most analyses. As comparison we used package DISMIR (https://github.com/XWangLabTHU/DISMIR, not versioned), CancerDetector (code obtained via the download form at https://zhoulab.dgsom.ucla.edu/pages/CancerDetector/, not versioned) and Houseman's deconvolution method (code available in the original publication https://doi.org/10.1186/1471-2105-13-86 , Supplementary File 2 https://static-content.springer.com/esm/art%3A10.1186%2F1471-2105-13-86/MediaObjects/12859_2012_5530_MOESM2_ESM.zip). DMR identification was performed using DSS v2.34.0.

For manuscripts utilizing custom algorithms or software that are central to the research but not yet described in published literature, software must be made available to editors and reviewers. We strongly encourage code deposition in a community repository (e.g. GitHub). See the Nature Portfolio guidelines for submitting code & software for further information.

Data

Policy information about availability of data

All manuscripts must include a data availability statement. This statement should provide the following information, where applicable:

- Accession codes, unique identifiers, or web links for publicly available datasets
- A description of any restrictions on data availability
- For clinical datasets or third party data, please ensure that the statement adheres to our policy

All data sets used in the study are downloaded from Gene Expression Omnibus (GEO). DLBCL WGBS data was downloaded with the accession number GSE137880, and ctDNA blood plasma samples (targeted BS-seq) were downloaded with the accession number GSE149438. The colorectal cancer (scBS-seq) and pancreatic cancer (WGBS) samples were downloaded with the accession numbers GSE97693 and GSE63123, respectively. All samples we downloaded from the normal cell atlas are available with the accession number GSE186458. We share processed read-level methylomes per patient for the lymph node WGBS data from prostate cancer patients as Supplemenatry Data.

Research involving human participants, their data, or biological material

Policy information about studies with human participants or human data. See also policy information about sex, gender (identity/presentation), and sexual orientation and race, ethnicity and racism.

Reporting on sex and gender	Not applicable and not reported
Reporting on race, ethnicity, or other socially relevant groupings	Not applicable and not reported
Population characteristics	Not applicable and not reported
Recruitment	Not applicable and not reported
Ethics oversight	Not applicable

Note that full information on the approval of the study protocol must also be provided in the manuscript.

Field-specific reporting

Please select the one below that is the best fit for your research. If you are not sure, read the appropriate sections before making your selection.

☒ Life sciences ☐ Behavioural & social sciences ☐ Ecological, evolutionary & environmental sciences

For a reference copy of the document with all sections, see nature.com/documents/nr-reporting-summary-flat.pdf

Life sciences study design

All studies must disclose on these points even when the disclosure is negative.

Sample size	Most experiments were performed in at least 15 experimentally measured samples or pseudobulk replicates. Given large effect sizes and low variance at studied DMRs this was sufficient to estimate the dispersion of MethylBERT solutions.
Data exclusions	No data excluded
Replication	Most experiments were performed in at least 15 experimentally measured samples or pseudobulk replicates.
Randomization	Separation into training, testing and validation sets was performed on a random sampling basis.
Blinding	Not applicable

Reporting for specific materials, systems and methods

We require information from authors about some types of materials, experimental systems and methods used in many studies. Here, indicate whether each material, system or method listed is relevant to your study. If you are not sure if a list item applies to your research, read the appropriate section before selecting a response.

Materials & experimental systems

n/a	Involved in the study
☒	☐ Antibodies
☒	☐ Eukaryotic cell lines
☒	☐ Palaeontology and archaeology
☒	☐ Animals and other organisms
☒	☐ Clinical data
☒	☐ Dual use research of concern
☒	☐ Plants

Methods

n/a	Involved in the study
☒	☐ ChIP-seq
☒	☐ Flow cytometry
☒	☐ MRI-based neuroimaging

Plants

Seed stocks	Not applicable
Novel plant genotypes	Not applicable
Authentication	Not applicable
